# Prospective observational study of oral clonazepam to prevent high-dose busulfan-induced seizures in adult patients

**DOI:** 10.1186/s43046-025-00257-3

**Published:** 2025-02-03

**Authors:** María Sacramento Díaz-Carrasco, Andrés Sánchez-Salinas, Juan José Fernández-Ávila, Raquel Olmos-Jiménez, Ignacio Español-Morales, Alberto Espuny-Miró

**Affiliations:** Hospital Universitario Virgen de La Arrixaca, Murcia, Spain

**Keywords:** Busulfan, Clonazepam, Seizures, Stem cell transplantation

## Abstract

**Background:**

Busulfan at high doses has been associated with a risk of seizures. Phenytoin has been used traditionally as anti-seizure prophylaxis, and benzodiazepines and levetiracetam have been introduced more recently, providing data from retrospective series. The main purpose of this study was to evaluate the effectiveness of oral clonazepam as anti-seizure prophylaxis in adult patients receiving high doses of intravenous busulfan as part of the conditioning regimen for hematopoietic stem cell transplantation. The secondary objectives were to determine the feasibility of this regimen and to analyze the adverse events associated with the use of clonazepam.

**Methods:**

This prospective, single-center study included 64 adult patients who received conditioning regimens with high doses of intravenous busulfan and anti-seizure prophylaxis with oral clonazepam, at a dose of 1 mg/8 h, from 12 h before starting treatment with busulfan until 48 h after ending administration.

**Results:**

The effectiveness of the prophylaxis was 100%, with no episodes of seizures during busulfan treatment or in the 72 h afterwards. Treatment was feasible, and oral scheduled administration was completed in all patients. Adverse events that could be associated with clonazepam included the onset of somnolence, dizziness, and confusion, mostly mild.

**Conclusion:**

The oral clonazepam regimen described in this study has been prospectively shown to be an effective, feasible anti-seizure prophylaxis option with manageable toxicity.

## Background

High-dose busulfan is frequently used in conditioning regimens for hematopoietic stem cell transplantation (HSCT). The drug can freely penetrate the blood–brain barrier, reaching concentrations in the central nervous system (CNS) similar to those of plasma [1, 2], causing neuronal damage and neurotoxic effects. One of its most relevant side effects is the onset of generalized tonic–clonic seizures, described in approximately 10% of patients (range 1.8–40%) when prophylactic measures are not used [3, 4]. Seizures usually appear between the second day of busulfan administration and 24 h after the last dose [3–5].

Historically, phenytoin has been the drug most widely used for prophylaxis of busulfan-induced seizures, decreasing its incidence to 0–5.5%; however, its use has been questioned due to its toxicity and potential for interactions [3, 5]. Phenytoin can cause bone marrow suppression on rare occasions; more frequently, it can cause an increase in liver enzymes and bilirubin, as well as skin rash. Signs and symptoms of these toxicities may overlap or be confused with toxicities inherent to the conditioning regimen, symptoms of hepatic sinusoidal obstructive syndrome (HSOS), or acute graft-versus-host disease (aGVHD). Moreover, phenytoin is capable of interacting with multiple components of the conditioning regimen. Oral busulfan has been specifically associated with increased clearance, while the results obtained with intravenous busulfan are contradictory [3].

Although benzodiazepines have generally been the drugs of choice for toxic seizures, their use as prophylaxis for busulfan-associated seizures has been suggested due to their low risk of hematologic, dermatologic, or hepatic toxicity and their minimal potential for pharmacokinetic interactions, although they increase other adverse effects, such as sedation. Pharmacokinetic studies have been conducted and showed no interactions between busulfan and various benzodiazepines [3, 6, 7]. Within the benzodiazepine group, clonazepam and lorazepam have the additional advantages of their short half-life and the absence of active metabolites [6]. There is more clinical experience in children [4, 7–10] than in adults [11], and the dosing regimens described are highly variable.

The use of other traditional anti-seizure medications, such as valproic acid and carbamazepine, is limited by their toxicity profile and interactions, especially the onset of rash that can overlap or mask the symptoms of cutaneous GVHD [3, 12]. More recently, the use of levetiracetam has been shown to be effective and safe in this setting, in small retrospective series [12–14].

Anti-seizure alternatives to phenytoin have been shown to be safe, without adversely affecting the risk of relapse, in a retrospective series involving more than 2000 patients undergoing allogeneic HSCT conditioned with busulfan and cyclophosphamide [15].

Intravenous clonazepam, at a fixed dose of 1 mg every 8 h, has been used as prophylaxis for busulfan-induced seizures. In 33 adult patients treated with this regimen, the effectiveness was 100% [16]. Since June 2012, it was decided to use this regimen at the center, administered orally, in line with the policy of preferential use of this route, if it is tolerated by the patient. Prospective follow-up of patient outcomes was also planned, in line with standard healthcare practice.

The main purpose of this study was to evaluate the effectiveness of oral clonazepam as anti-seizure prophylaxis at a fixed dose of 1 mg every 8 h in adult patients receiving high doses of intravenous busulfan (IV BU) as part of the conditioning regimen for HSCT. The secondary objectives were to determine the feasibility of this regimen in patients undergoing aggressive conditioning treatments, which can reduce oral tolerance, and to analyze the adverse events associated with the use of clonazepam.

## Methods

**Fig. 1:**
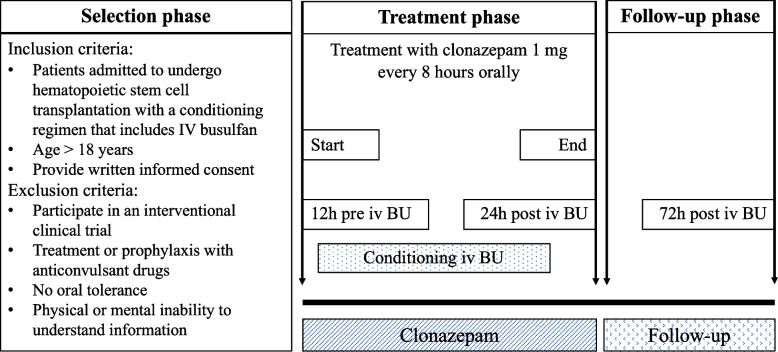
Study design

An observational, non-interventional, prospective, single-center, prospective follow-up study was designed, which included adult patients undergoing HSCT, with a conditioning regimen including IV BU, in a 900-bed university hospital. The inclusion and exclusion criteria are shown in Fig. 1.

Patients included in the study were monitored according to standard clinical practice for the duration of the study. The prospective follow-up period included two phases, which are also shown in Fig. 1.

1. Treatment phase: The entire period the patient is receiving oral clonazepam, from 12 h before the start of the IV BU to 24 h after end of treatment.
2. Follow-up phase: Until 48 h after the end of oral clonazepam administration (72 h after the end of IV BU administration).

The inclusion period was from May 2014 to December 2022.

The following independent variables were recorded: demographic data (age and gender), clinical data (diagnosis motivating HSCT, previous lines), history of neurological disorders, and patient’s performance status according to the Eastern Cooperative Oncology Group (ECOG) [17], variables related to HSCT (type of transplant, cell source, and type of donor) and conditioning regimen (drugs, doses, days), and concomitant medication received by the patient.

The primary endpoint of the study was the effectiveness of treatment with oral clonazepam, defined as the percentage of patients who were seizure-free from the start of treatment with IV BU until 72 h after the end of administration. Other dependent variables recorded were clonazepam treatment feasibility, defined as the percentage of patients who tolerated oral clonazepam treatment, and adverse events observed during treatment, classified according to CTCAE v4.0 criteria [18].

The sample size was calculated according to the primary endpoint, the percentage of patients undergoing seizures after receiving busulfan. Given that the percentage described in the literature [3, 4] is 10% and considering a statistical power of 80% and a significance level of 5%, 58 patients are required. Considering a sample loss of 10%, 64 patients are required.

The study was conducted in accordance with the guidelines for Good Epidemiological Practice [19]. Local ethical committee authorization and informed consent from all included patients were obtained.

## Results

During the study period, 71 patients received busulfan conditioning, of whom 7 were excluded from the study for the following reasons: there were 4 patients who received gabapentin or pregabalin for neuropathic pain or persistent hiccups, 2 were included in interventional clinical trials, and 1 patient started clonazepam, by mistake, outside the window established in the study. The study included 64 patients, of whom 65.6% were male, with a median age of 53 years, whose characteristics are shown in Table 1. This table also includes the characteristics of the HSCT received (autologous or allogeneic), the donor (related, unrelated, haploidentical), the source of stem cells (peripheral blood or bone marrow), and the drugs received during conditioning.

**Table 1:** Demographic, clinical variables and variables related to the type of hematopoietic stem cell transplant and the conditioning received

Patient characteristics		Treatment characteristics	
**Number of patients**	64	**Type of transplant**	
**Age** Median (range)	53 (22–69)	• Autologous	11
		• Allogenic	53
**Sex** Female Male	22 42	o Related donor	20
		o Unrelated donor	14
		o Haploidentical	19
**Diagnosis**		**Stem cells source**	
Acute myeloid leukemia	31	• Peripheral blood	63
Myelodysplastic syndrome	13	• Bone marrow	1
Multiple myeloma	4	**Busulfan dose**	3.2 mg/kg/day
Myelofibrosis	4	**Duration (days)**	2–4
Diffuse large B-cell lymphoma	2	• 2	16a
Mantle cell lymphoma	2	• 3	15
Acute lymphoblastic leukemia	2	• 4	33
Chronic myeloid leukemia	2	**Drugs associated with IV BU in the conditioning regimen**	
Chronic myelomonocytic leukemia	2	• Fludarabine	20
Follicular lymphoma	1	• Fludarabine-thymoglobulin	13
Acute promyelocytic leukemia	1	• Fludarabine-thiotepa	15
**ECOG**		• Etoposide-cytarabine	6
0	50	• Melphalan	4
1	13	• Fludarabine-cyclophosphamide	3
2	1	• Cyclophosphamide	2
**Number previous lines** Median (range)	1 (0–4)	• Fludarabine-thiotepa-thymoglobulin	1
		**Triple intrathecal treatment** (MTX 12 mg/ARAC 30 mg/HC 20 mg)	
		• No	33
		• Yes	31b

*ARAC *cytarabine, *IV BU *intravenous busulfan, *HC *hydrocortisone, *MTX *methotrexate

^a^One patient took 3 mg/kg/day for 2 days due to liver toxicity

^b^One double IT case, without MTX

Two of the 64 patients included had a clinical history of neurological events. Both had previously suffered a stroke: one patient 4 years before HSCT and the other 1 year prior to HSCT; the former patient was still experiencing mild right hemiparesis. None of the patients had a history of seizures.

The autologous transplantations were performed on six patients with acute myeloid leukemia (AML), conditioned with BEA regimen, one patient with acute promyelocytic leukemia with BuCy2, and four patients with multiple myeloma conditioned with BUMEL regimen (Table 2). The specific cases of allogeneic transplants were more variable; transplants from related and haploidentical donors were predominant, with AML being the most frequent diagnosis and busulfan with fludarabine the most commonly used combination in conditioning. ATG (Timoglobulina™) was included in conditioning, when the donor was unrelated, and six patients received low doses of total body irradiation (TBI) (Table 2).

**Table 2:** Preparative regimens used for HSCT conditioning

Preparative regimen	Regimen	Days	N°
BEA-BU (12.8)/VP16 (40)/ARAC (12,000)	Busulfan 3.2 mg/kg/day Etoposide 20 mg/kg/day Cytarabine 3000 mg/m^2^ c/12 h	4 (− 8, − 7, − 6, − 5) 2 (− 4, − 3) 2 (− 3, − 2)	6
BuCy2 BU (12.8)/CFM (120)	Busulfan 3.2 mg/kg/day Cyclophosphamide 60 mg/kg/day	4 (− 8, − 7, − 6, − 5) 2 (− 3, − 2)	2
BUMEL-BU (9.6)/MEL (140)	Busulfan 3.2 mg/kg/day Melphalan 140 mg/m^2^/day	3 (− 5, − 4, − 3) 1 (− 1)	4
FLU (150)/BU (9.6)/CFM (29)	Fludarabine 30 mg/m^2/day^ Busulfan 3.2 mg/kg/day Cyclophosphamide 14.5 mg/kg/day	5 (− 6, − 5, − 4, − 3, − 2) 3 (− 4, − 3, − 2) 2 (− 6, − 5)	2
FLU (160)/BU (12.8)	Fludarabine 40 mg/m^2^/day Busulfan 3.2 mg/kg/d	4 (− 6, − 5, − 4, − 3) 4 (− 6, − 5, − 4, − 3)	15
FLU (150)/BU (6.4)-ICT 2 Gy	Fludarabine 30 mg/m^2/day^ Busulfan 3.2 mg/kg/day TBI 2 Gy	5 (− 6, − 5, − 4, − 3, − 2) 2 (− 6, − 5) or (− 3, − 2) 1 (− 1)	5
FLU (150)/BU (6.4)/ATG (6)	Fludarabine 50 mg/m^2^/day Busulfan 3.2 mg/kg/day ATG (rabbit) 2 mg/kg/day	3 (− 6, − 5, − 4) 2 (− 6, − 5) 3 (− 4, − 3, − 2)	1
FLU (150)/BU (6.4)/CFM (29)-ICT 2 Gy	Fludarabine 30 mg/m^2^/day Busulfan 3.2 mg/kg/day Cyclophosphamide 14.5 mg/kg/day TBI 2 Gy	5 (− 6, − 5, − 4, − 3, − 2) 2 (− 4, − 3) 2 (− 6, − 5) 1 (− 2)	1
FLU (160)/BU (12.8)/ATG (6)	Fludarabine 40 mg/m^2^/day Busulfan 3.2 mg/kg/day ATG (rabbit) 2 mg/kg/day	4 (− 6, − 5, − 4, − 3) 4 (− 6, − 5, − 4, − 3) 3 (− 3, − 2, − 1)	10
FLU (160)/BU (9.6)/ATG (6)	Fludarabine 40 mg/m^2^/day Busulfan 3.2 mg/kg/day ATG (rabbit) 2 mg/kg/day	4 (− 6, − 5, − 4, − 3) 3 (− 6, − 5, − 4) 3 (− 3, − 2, − 1)	2
TIOT (10)/FLU (150)/BU (6.4)	Thiotepa 5 mg/kg/day Fludarabine 50 mg/m^2^/day Busulfan 3.2 mg/kg/day	2 (− 6, − 5) 3 (− 4, − 3, − 2) 2 (− 4, − 3)	7
TIOT (10)/FLU (150)/BU (6.4)/MP (25)	Thiotepa 5 mg/kg/day Fludarabine 30 mg/m^2^/day Busulfan 3.2 mg/kg/day Methylprednisolone 5 mg/kg/day	2 (− 3, − 2) 5 (− 6, − 5, − 4, − 3, − 2) 2 (− 6, − 5) 5 (− 6, − 5, − 4, − 3, − 2)	1
TIOT (10)/FLU (150)/BU (9.6)	Thiotepa 5 mg/kg/day Fludarabine 50 mg/m^2^/day Busulfan 3.2 mg/kg/day	2 (− 6, − 5) 3 (− 4, − 3, − 2) 3 (− 4, − 3, − 2)	6
TIOT (10)/FLU (125)/BU (6.4)/MP (25)	Thiotepa 5 mg/kg/day Fludarabine 25 mg/m^2^/day Busulfan 3.2 mg/kg/day Methylprednisolone 5 mg/kg/day	2 (− 3, − 2) 5 (− 6, − 5, − 4, − 3, − 2) 2 (− 6, − 5) 5 (− 6, − 5, − 4, − 3, − 2)	1
TIOT (10)/FLU (150)/BU (9.6)/ATG (6)	Thiotepa 5 mg/kg/day Fludarabine 50 mg/m^2^/day Busulfan 3.2 mg/kg/day ATG (rabbit) 2 mg/kg/day	2 (− 6, − 5) 3 (− 4, − 3, − 2) 3 (− 4, − 3, − 2) 3 (− 4, − 3, − 2)	1

*ATG* antithymocyte globulin. Timoglobulina™, *TBI* total body irradiation

None of the patients received HSCT from umbilical cord blood, and bone marrow was used as a source of stem cells in only one procedure, from an unrelated donor, in a patient with myelodysplastic syndrome conditioned with FluBuATG.

Busulfan was administered at a dose of 3.2 mg/kg every 24 h, except for one patient who received a reduced dose of 3 mg/kg every 24 h due to previous liver toxicity. Treatment duration was 2–4 days depending on the desired intensity of the conditioning regimen. The conditioning regimens are described in detail in Table 2.

During the study period, between 94 and 100% of patients received ondansetron, ursodeoxycholic acid, omeprazole, acyclovir, co-trimoxazole, enoxaparin, allopurinol, and furosemide. Additionally, 55 of the 64 patients (85.9%) were prescribed lorazepam (53 patients) (1 mg daily) or alprazolam (2 patients) as a hypnotic; on 14 occasions (21.9%), the benzodiazepine was administered concomitantly with clonazepam and in the remaining patients after discontinuing administration of clonazepam. The following drugs were also frequently prescribed (between 25 and 60% of patients): fluconazole, dexchlorpheniramine, rifaximin, dexamethasone, sodium bicarbonate, paracetamol, meropenem, metoclopramide, tacrolimus, lactulose, metamizole, and methylprednisolone.

Effectiveness was 100%; no patient experienced seizure episodes during IV BU treatment or for 72 h afterwards.

All patients completed treatment with oral clonazepam. The planned treatment was administered in 62 patients (96.9%) without any changes, with the exception of 2 patients whose dosage was reduced by 50% due to moderate symptoms of drowsiness and dizziness.

Adverse events were recorded in 60 patients during the study period, as shown in Table 3.

**Table 3:** Adverse events recorded during the study period

Adverse event	*n*	%	Grade			
			**1**	**2**	**3**	**4**
Somnolence	27	42.19	18	8	1	
Nausea	25	39.06	17	7	1	
Hyperglycemia	25	39.06	11	8	5	1
Vomiting	24	37.5	22	1	1	
ALT/AST increased	18	28.13	14	2	2	
Headache	15	23.44	11	4		
Dizziness	12	18.75	10	2		
Diarrhea	11	17.19	10	1		
Fever	8	12.5	5	3		
Confusion	8	12.5	7	1		
Mucositis	6	9.38	6			
Constipation	6	9.38	6			
Fatigue	6	9.38	6			
Hypotension	3	4.69		1	2	
Rash	3	4.69		2	1	
Creatinine increased	2	3.13	2			
Hypertension	2	3.13		1	1	
Back pain	2	3.13	2			
Anaphylaxis	2	3.13		1	1	
Chills	2	3.13	1	1		
Dysphagia	1	1.56	1			
Ataxia	1	1.56	1			
Hypothermia	1	1.56		1		
Cognitive disturbance	1	1.56		1		
Sepsis	1	1.56				1
Pain in extremity	1	1.56		1		
Superficial thrombophlebitis	1	1.56		1		
Palpitations	1	1.56		1		
Bone pain	1	1.56	1			
Blood bilirubin increased	1	1.56	1			
Presyncope	1	1.56		1		
Hyponatremia	1	1.56	1			

*ALT *alanine aminotransferase, *AST *aspartate aminotransferase

Grade 1, mild; 2, moderate; 3, severe; 4, life-threatening consequences

Adverse events with the highest probability of association with clonazepam administration were as follows:

- Somnolence in 27 patients (42.2%), mostly mild, with only one case severe. In the latter case, chlorpromazine was concomitantly administered for persistent hiccups. Dexchlorpheniramine was concomitantly administered in six cases, four of which were moderate and two of which were mild, while in another mild case the patient requested and received a dose of lorazepam 1 mg. One patient underwent moderate drowsiness after discontinuation of clonazepam, coinciding with the administration of dexchlorpheniramine.
- Dizziness mild-moderate appeared in 12 patients (18.7%), of which 2 cases were associated with dexchlorpheniramine.
- Confusion was observed in eight patients (12.5%), with a single case of moderate confusion in a patient with other associated factors (cerebral lymphoma with stable disease at the date of transplantation, thiotepa administered at conditioning, and ECOG 2). There was one mild case described after discontinuation of clonazepam, coinciding with the prescription of an antidepressant.

The remaining adverse events listed in Table 3 were most likely associated with other drugs administered during conditioning.

## Discussion

To the authors’ knowledge, we present the results of the largest prospective series to date, with 64 patients, for the evaluation of seizure prophylaxis other than phenytoin in patients treated with high-dose busulfan. The effectiveness was 100%, similar to the previous study with the same drug administered by IV route [16], and higher than historical data with phenytoin, which establish seizure frequency at between 0 and 5.5% [3]. The clonazepam treatment period ranged from 2.5 to 5.5 days, with the specific duration dependent on the number of days of busulfan administration. The absence of seizures over the course of treatment suggests that tachyphylaxis did not occur.

Comparison of results with other studies is difficult since prophylaxis doses, times, and intervals, as well as busulfan doses and route of administration, vary greatly.

In a retrospective, multicentric series that includes 954 HSCT procedures in pediatric patients, 66% of which were treated with oral busulfan and 34% of which were treated with IV busulfan, Caselli et al. [20] describe a seizure incidence of 1.3%, with 0.52% attributed by the authors to the drug. In this study, all the patients received prophylaxis with a high variety of drugs and dosing regimens (carbamazepine, clonazepam, valproate, phenobarbital, lorazepam, etc.).

Compared to other studies with benzodiazepines, it is worth mentioning the review by Eberly [3] which includes several studies with different benzodiazepines and different doses and dosing regimens; the study reviews small series (8–46 patients), mainly pediatric, with seizure results varying between 0 and 4.5% of patients (note that in the two studies reporting seizures, one case was that of a child who had not received the intended benzodiazepine and the other of a patient who had a history of seizures and was receiving concomitant carbamazepine). Carreras et al. [6] used clonazepam IV, administered in continuous perfusion, in 24 adult patients, with the aim of studying the possible influence of this prophylaxis on the pharmacokinetics of IV busulfan. No cases of seizures occurred in their study. On the other hand, Tsujimoto et al. [21] retrospectively compared clonazepam (*n* = 13) with levetiracetam (*n* = 30) in children. In their study, they describe no cases of seizures in the clonazepam-treated series, while there were two cases in the levetiracetam group (6.7%). The differences are not statistically significant, given the small sample size.

Other studies using levetiracetam prophylaxis do not describe seizures: Akiyama et al. [13] did not observe any cases in 34 adult patients treated with levetiracetam, while 1 patient out of 70 treated with phenytoin developed seizures in their retrospective series. Similarly, Nakashima et al. [12] did not describe any cases of seizures in a series of 46 adult patients treated with levetiracetam, neither did Chaguaceda et al. [14] in 36 adult patients receiving 1000 mg every 12-h doses of levetiracetam nor did Floeter et al. [22] in 9 pediatric patients, or Soni et al. [23] in a cohort of 28 children and young adults, using IV levetiracetam. These series, although all of them retrospective, reported reasonable outcomes.

Although the use of prophylaxis in this context is widely established, some authors question its use [24], arguing that there are also data on the occurrence of seizures despite the use of prophylaxis [5, 20] and based on their experience of minimal incidence without prophylaxis (0.01%, 1 case out of 96), in a retrospective series, with busulfan administered orally. Ruiz-Arguelles et al. [25] also question the need for anti-seizure prophylaxis in all conditioning settings; in their experience with 344 patients conditioned with a reduced-intensity regimen (oral busulfan 8 mg/kg over 2 days), they observed no cases of seizures.

Tolerance of the scheduled oral clonazepam regimen was very good, so the oral route was maintained in 100% of the patients, and it was only necessary to reduce the dose in 3.1% of patients, due to the clinical criterion of excessive drowsiness, albeit classified as moderate.

Most adverse events described during the follow-up period were frequently associated with other conditioning and supportive therapy components that are well-known for their toxicity profile: nausea and vomiting associated with chemotherapy, hyperglycemia associated with the use of corticosteroids, transaminitis associated with busulfan or fluconazole, or reactions associated with ATG infusion.

All benzodiazepines are associated with sedation and poor coordination. None of these is commonly associated with hematological, dermatological, or hepatic toxicity [3]. The adverse events most likely to be associated with to clonazepam were somnolence (40.6%), dizziness (18.8%), and confusion (10.9%), mostly mild. It is worth noting the involvement in these events of other CNS depressant drugs prescribed simultaneously, albeit on an ad hoc basis, particularly dexchlorpheniramine and chlorpromazine. The use of these combinations should be limited, and patients should be closely monitored if their administration is essential. One caution that should also be considered with clonazepam is the potential for falls and stumbles due to somnolence, vertigo, and decreased muscle tone, although these were not reported in this study.

Other studies do not present a comprehensive description of adverse events during the period of prophylaxis administration, but focus on how prophylaxis affects typical conditioning toxicity (HSOS, oral mucositis, pulmonary toxicity, etc.) [13, 23], or describe the toxicities to be expected with the drug used in prophylaxis [12, 14, 21], in particular neurological events. Tsujimoto et al. [21] describe more frequent somnolence (46%) and dizziness (54%), together with 62% irritability, in their pediatric population treated with clonazepam, while they observed less, but not statistically significant, somnolence with levetiracetam (20%), with no cases of irritability or dizziness in this group. Nakashima et al. [12] describe 19.5% somnolence with levetiracetam, with no indication of the grade; Chaguaceda et al. [14] indicate that the most frequent adverse effects are somnolence, without specifying a percentage, and irritability (11.1%, without mentioning the grade), while Soni et al. [23] only report that they did not observe excessive somnolence or behavioral changes. Other studies have described percentages of somnolence around 50% with lorazepam [7], 9% with phenytoin, or 12.5% with valproic acid [12].

This study is mainly limited by its single-center and non-comparative nature, as well as by a range of conditioning of different intensities, which could be associated with various levels of seizure risk [25]. Plasma busulfan levels were not monitored, as in most published studies [12–14, 16, 24, 25], although monitoring could reduce the risk of this and other toxicities [2, 26]. Another suggested option is the inclusion of pharmacogenetic studies to guide dosing, with the aim of increasing the effectiveness and reducing the toxicity of high-dose busulfan. However, this practice has not yet been standardized [27].

## Conclusion

The oral clonazepam regimen described in this study has been prospectively shown to be an effective, feasible anti-seizure prophylaxis option with manageable toxicity.

## Data Availability

Data is provided within the manuscript.

## Abbreviations

aGVHD: Acute graft-versus-host disease
AML: Acute myeloid leukemia
ATG: Antithymocyte globulin
CNS: Central nervous system
GVHD: Graft-versus-host disease
HSCT: Hematopoietic stem cell transplantation
HSOS: Hepatic sinusoidal obstructive syndrome
IV BU: Intravenous busulfan
TBI: Total body irradiation
